# Biostimulant Effect and Biochemical Response in Lettuce Seedlings Treated with A *Scenedesmus quadricauda* Extract

**DOI:** 10.3390/plants9010123

**Published:** 2020-01-18

**Authors:** Ivana Puglisi, Emanuele La Bella, Ermes Ivan Rovetto, Angela Roberta Lo Piero, Andrea Baglieri

**Affiliations:** Dipartimento di Agricoltura, Alimentazione e Ambiente (Di3A), Università di Catania, Via S. Sofia 98, 95123 Catania, Italy; emanuelelabella95@gmail.com (E.L.B.); ermes.rovetto@hotmail.com (E.I.R.); rlopiero@unict.it (A.R.L.P.); abaglie@unict.it (A.B.)

**Keywords:** chlorophylls, GOGAT, glutamine synthetase, citrate sintase, malate dehydrogenase, PAL

## Abstract

The use of natural biostimulants is becoming an attractive option in order to reduce the use of fertilizer and increase the yield of crops. In particular, algal extracts are suitable candidates as they positively affect plant physiology. Among crops, lettuce often requires the use of biostimulants to improve both the quality and quantity of production. The aim of this work is to investigate the potential use of a *Scenedesmus quadricauda* extract as a biostimulant in order to obtain sustainable cultivation and a reduction in the cost of chemical fertilizers in lettuce cultivation. Therefore, the effect of *S. quadricauda* extract on lettuce seedlings was explored by evaluating the physiological parameters, chlorophyll, carotenoid, and total protein contents as well as several plant enzymatic activities involved in primary and secondary metabolisms. The experiment was performed by growing plants on inert substrate (pumice) with a 16-h photoperiod, by carrying out two consecutive radical treatments, one week apart, using a concentration of the extract corresponding to 1 mg Corg L^−1^. Lettuce plants were sampled at 1, 4, and 7 days from the first treatment and 7 days from the second treatment. The results showed that the *S. quadricauda* extract positively affected the growth of lettuce seedlings, mainly acting at the shoot level, determining an increase in dry matter, chlorophylls, carotenoids, proteins, and influencing the activities of several enzymes involved in the primary metabolism.

## 1. Introduction

The interest in the production of microalgae and their cultivation has recently increased worldwide due to their great economic and commercial relevance as well as for their wide fields of application [1,2]. Microalgae biomasses are used to produce biofuels and biomaterials, and their byproducts are used in the food and feed sectors [3]. Several studies have been focused on their production and economic yield. Microalgae are photosynthetic organisms of great interest due to their adaptation to different cultivation systems, being autotrophic, heterotrophic, or mixotrophic [1].

In order to attain sustainable and environmentally friendly agricultural systems, the use of plant natural biostimulants nowadays is always increasing. These compounds are a wide range of molecules able to promote plant growth if applied to the soil in small quantities [4]. Biostimulants positively affect plant growth by enhancing water uptake, root and shoot growth, tolerance to abiotic stress, protein content in plant tissues, and the activity of the enzymes connected to the assimilation of nitrogen and photosynthesis [4,5,6,7,8,9]. The biostimulant action also includes an increase in the activity of key enzymes involved in carbon metabolism [10,11], the enhancement of hormone-activity [12,13], and physiological, biochemical and anatomical changes such as the production of antioxidant enzymes, pigments, and secondary metabolites [13,14,15].

Among biostimulant compounds, seaweed extracts have shown to positively affect the physiology of plants by influencing both the transcriptome and metabolome profiles of the treated plants [15,16,17]. Fan et al. [18] found that spinach seedlings treated with a commercial brown algal extract increased in transcription of gene coding regulatory enzymes involved in the nitrogen metabolism and in the antioxidant regulatory system, which was associated with a boost of total protein as well as the phenolic and flavonoid contents.

It was shown that extracts from the microalgae *C. vulgaris* and *S. quadricauda,* applied to the Hoagland solution, exerted a biostimulant effect on sugar beet at its early stages of growth [19]. Similarly, these same extracts from *C. vulgaris* and *S. quadricauda*, directly applied into the soil, increased the growth parameters in tomatoes [20]. Microalgae secrete a large number of biologically active extracellular molecules, indicated as secondary metabolites, also known as allelochemicals [21]. The same molecules produced by living microalgae exert a biostimulant effect on plants, as shown by Barone et al. [22], who found a biostimulant effect on tomato plants in a co-cultivation system between tomato and microalgae (*S. quadricauda* or *C. vulgaris*).

Lettuce (*L. sativa* L.) is one of the most important vegetable crops grown in the Mediterranean area; however, it is a moderately sensitive crop to salt, therefore the use of biostimulant is required as a very useful practice to enhance yield [23]. Kopta et al. [24] found that a preparation based on microalgae (*C. vulgaris*) and plant growth-promoting bacteria (*Bacillus licheniformis*, *Bacillus megatherium*, *Azotobacter* sp., *Azospirillum* sp., and *Herbaspirillum* sp.) positively affected the yield and nutritional parameters of lettuce cultivated as a spring and summer crop by increasing the fresh weight, the total antioxidant capacity, and total carotenoid content.

In view of the worldwide trend focusing on the biotechnology production of microalgae bioproducts to use in sustainable agriculture, the aim of this work is to evaluate the effect of an extract from *S. quadricauda* on lettuce seedling growth as well as the plant biochemical response to the treatment. Consequently, the physiological parameters of leaf and root were monitored. Moreover, the effect of the extract on the plants was also evaluated by monitoring the activities of enzymes involved in the nitrogen (glutamate synthase and glutamine synthase) and carbon (citrate synthase and malate dehydrogenase) metabolisms as well as of a key enzyme (phenylalanine ammonia lyase) implicated in the secondary metabolism. Finally, the levels of leaf proteins, chlorophylls, and carotenoid were also measured. Based on our results, the use of microalgal extracts as biostimulants might be a cheap method to obtain sustainable cultivation and a reduction in the cost of chemical fertilizers.

## 2. Results and Discussion

### 2.1. Physiological Parameters of Lettuce Seedlings

The growth of lettuce seedlings was monitored at each sampling time (as detailed in the Materials and Methods) in order to evaluate the effect of the treatment with *S. quadricauda* extract (SQ) as well as its effectiveness over the entire experimental period. The shoot height, root length, root weight, number of leaves of the plant, and total plant weight are reported in Table 1. The treatment positively affected total plant weight soon after four days from the first application (T4 (I)), hence keeping higher values than the control over the entire experimental period. It is interesting to note that the SQ treatment influenced all the parameters, mainly at T4 (I), except root weight. As regards the T7 (I) sampling, the root length of treated plants was significantly higher than the control, whereas, at all other sampling times, values were rather similar to the control (Table 1). These findings are in accordance with Barone et al. [19], who found that in the early stages of sugar beet plant growth, the addition of the *S. quadricauda* extract significantly increased the total root length, root surface area, and the number of root tips compared with the control plants.

**Figure 1:**
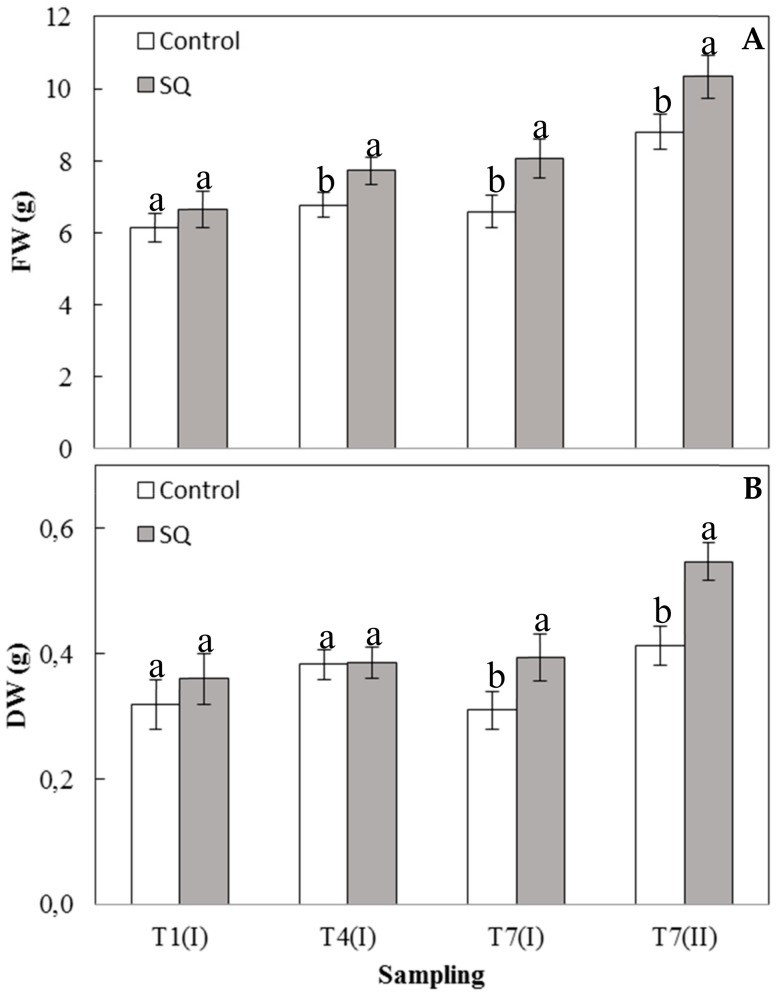
Fresh (**A**) and dry (**B**) weight (g) of shoots of lettuce seedlings. Error bars indicate standard deviation. The values are means of data from five replications. Values followed by different letters are significantly different (*p* < 0.05).

In order to deeply investigate the effect of the treatment on the weight of the lettuce seedlings and therefore on the effective yield, the fresh and dry weights of aerial portions of the plants, corresponding to the edible portion, were determined (Figure 1). SQ treatment increased the leaf fresh weight of seedlings at the beginning from T4 (I) until T7 (II) samplings (Figure 1A), according to data reported in Table 1. Noteworthy, the leaf dry weights were positively affected by the treatment at T7 (I) and T7 (II), the latter reaching an increase of around 26% in dry weight compared to the control (Figure 1B). Similarly, tomato plants, grown in pots of soil for 18 days and treated with the SQ extract at the concentration of 1 mg Corg L^−1^, increased their leaf dry weight of around 25% with respect to the control [20].

These results suggest that the treatment with the extract of *S. quadricauda* shows a biostimulant effect on lettuce seedlings and determines a greater influence at the shoot level, allowing the treated plants to accumulate a higher quantity of dry matter than control plants.

### 2.2. Protein and Pigment Contents

**Figure 2:**
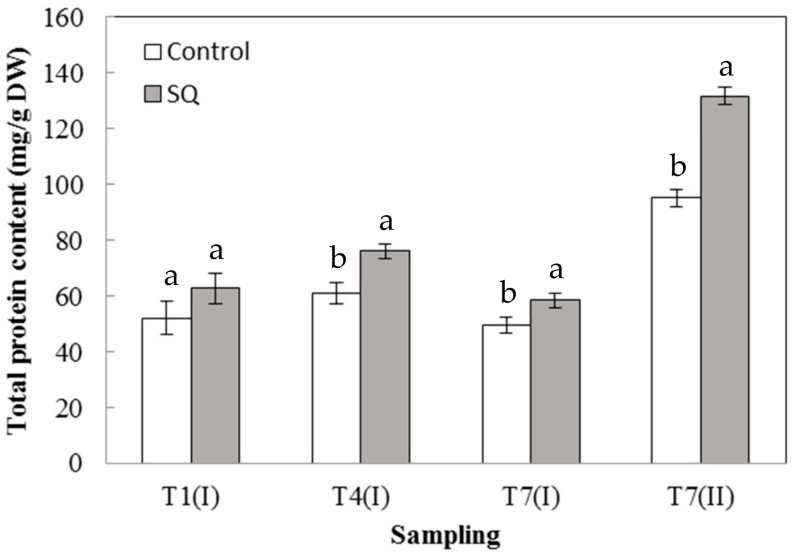
Total protein content in leaves of lettuce seedlings. Error bars indicate standard deviation. The values are means of data from five replications. Values followed by different letters are significantly different (*p*< 0.05).

Figure 2 shows the content of the total proteins extracted from the shoot of lettuce seedlings. Interestingly, the total protein content was strongly influenced by the treatment, as 4 days after the first treatment (T4 (I)), it increased to reach, at the end of the experimental period (T7 (II)), a value of around 38% higher than that measured in the control plants (Figure 2). The increase in total proteins is probably to cope with the increased growth of plants subjected to the treatment. Therefore, in order to deal with the increased protein biosynthesis, the plant must consequently increase the uptake of the nutrients at the root level. Our results are in accordance with Fan et al. [18], who found that in spinach treated with a commercial algae-based extract, an increase of total soluble proteins occurred and it was closely associated with an increase in the transcription level of regulatory enzymes involved in nitrogen metabolism.

The contents of chlorophyll a, chlorophyll b, and carotenoids are reported in Table 2. Interestingly, chlorophyll a and carotenoids sharply increased, with respect to the control, 1 day after the first treatment. All pigments, at all sampling times, showed values always significantly higher than the control, except chlorophyll b at T1 (I) recording a value similar to the control (Table 2). These results are in accordance with other findings on a wide range of crops, including grapevine and strawberry, in which an increase in chlorophyll contents was observed in those plants treated with algae extracts [18,25]. The pigments present in the antenna complex are mainly made up of chlorophylls b, xanthophylls and carotenoids, whereas chlorophyll a is known to be the core pigment in the reaction center [26,27]. Therefore, the treatment with SQ extract determines an increase of chlorophyll a and, thus, likely enlarging the photosystem number. Furthermore, an increase in the accessory pigments (chlorophyll b and carotenoids) was also observed (Table 2), thus, increasing the area of the molecules able to intercept the light and to transfer the absorbed energy to the reaction center.

These results suggest that the increase in the fresh and dry weights of treated lettuce seedlings (Figure 1) was probably due to an accumulation of soluble compounds in the leaves, such as proteins (Figure 2) and pigments (Table 2). Indeed, it is well known that the interception of solar radiation is strictly related to the increase in yield in the most important crops and the improvement in carbon fixation is an essential factor for the increase in the biomass as well as in the crop yield [28].

### 2.3. Enzyme Activities in Lettuce Seedlings

In order to investigate the effect of *S. quadricauda* extract on lettuce seedling metabolism, the activities of some key enzymes involved in the nitrogen primary metabolism (GOGAT and GS), the carbon primary metabolism (CS and MDH), and the secondary metabolism (PAL) were monitored.

**Figure 3:**
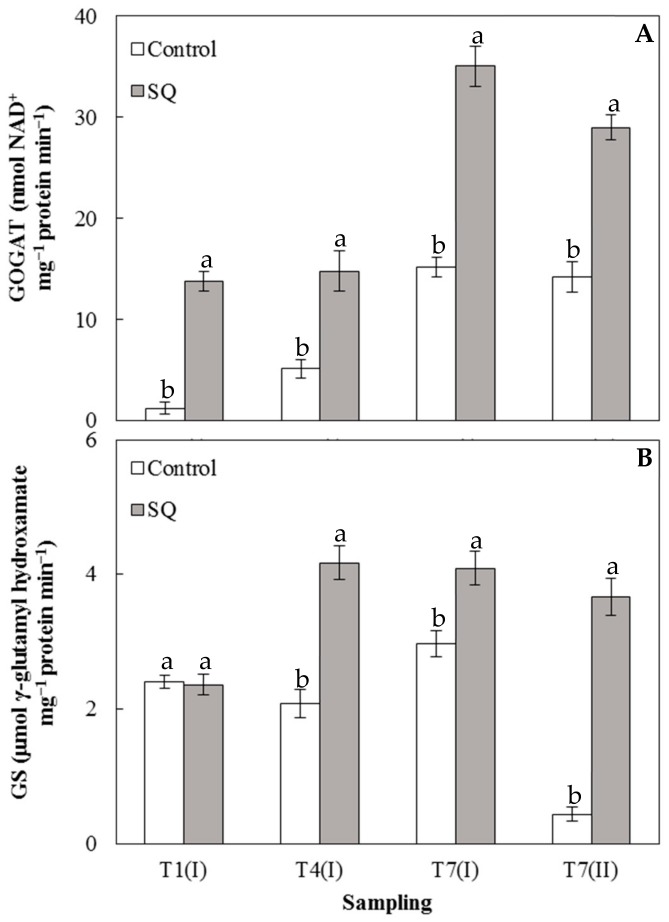
Glutamate synthase (GOGAT) activity (**A**) and glutamine synthase (GS) activity (**B**) in leaves of lettuce seedlings. Error bars indicate standard deviation. The values are means of data from five replications. Values followed by different letters are significantly different (*p* < 0.05).

Figure 3A shows the GOGAT activity measured in leaves of lettuce over the experimental period. At all the sampling times in the treated plants, an enzymatic activity significantly higher than the control was always recorded. The application of the SQ extract rapidly induced the activation of GOGAT, reaching at T1 (I) the highest value, around 11 times greater than that measured in the control. Similarly, in Figure 3B, it is shown that GS activity was always significantly higher in the treated plants than in the control at all the sampling times except at T1 (I), in which the activities were similar between treated and untreated plants. At the end of the experimental period (T7(II)), GS activity in treated plants were increased by around 8 times with respect to the control (Figure 3B). These results suggest that the positive effect exerted by the treatment on the growth of lettuce seedlings most likely occurs through the stimulation of the nitrogen metabolism. In fact, the isoenzymes of GOGAT and GS have been proposed to play an important role in the ammonium assimilation processes such as primary nitrogen assimilation [29,30]. Actually, ammonia is assimilated into organic form as glutamine and glutamate, acting as the nitrogen donors in the biosynthesis of amino acids, nucleic acids, and other nitrogen compounds such as chlorophylls [29,30]. According to previous results, a greater nitrogen absorption causes an increase in total proteins (Figure 2) as well as in photosynthetic pigments, which increase very fast (Table 2) and thus contribute to enhance the values of fresh and dry weights in the treated seedlings (Figure 1). These results are consistent with other studies, mainly carried out on maize, which demonstrated the ability of biostimulants to increase the GOGAT and GS activities [6,11].

**Figure 4:**
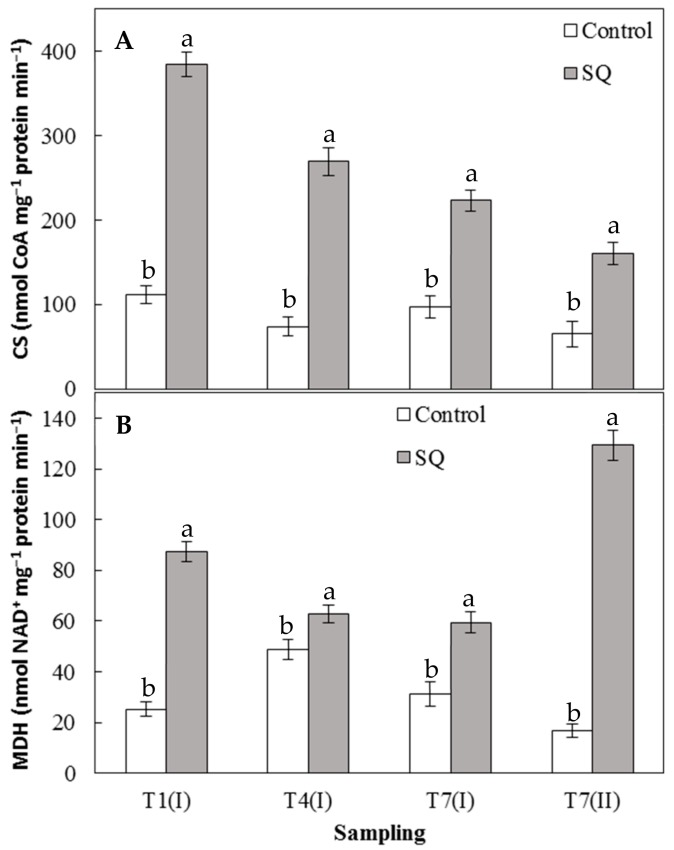
Cytrate synthase (CS) activity (**A**) and malate dehydrogenase (MDH) activity (**B**) in leaves of lettuce seedlings. Error bars indicate standard deviation. The values are means of data from five replications. Values followed by different letters are significantly different (*p* < 0.05).

The monitoring of CS activity is shown in Figure 4A. As observed with enzymatic activities involved in nitrogen metabolism, the treatment with the extract of *S. quadricauda* significantly increased all the values of CS activity when compared to untreated plants. Treatment appears to act on CS activity to a greater extent after the first treatment, inducing at T1 (I) and T4 (I) an increase of around three times when compared to the control (Figure 4A). At all other sampling times, CS activity in the treated lettuce seedlings decreased when compared to the T1 (I) samples; nevertheless, values were always significantly higher than those measured in control plants (Figure 4). These results suggest that the activation of CS occurs soon after the first application of the extract, supporting the hypothesis that CS may represent one of the key enzymes induced by the treatment. Moreover, Figure 4B shows that the SQ extract also positively affects the values of MDH activity, reaching, at the end of the experimental period (T7 (II)), the highest value with respect to the control (around seven times).

These results are very interesting, as the treatment induced both the nitrogen metabolism as well as the respiratory metabolism of leaf cells. In fact, CS represents the most important key enzyme of the Krebs cycle because it catalyzes the reaction that controls the rhythm of the respiratory tract [26,31]. Accordingly, a protein hydrolysate functioning as a biostimulant promoted nitrogen assimilation and shoot biomass production, soluble sugar accumulation, and nitrogen assimilation in maize. This was achieved via a coordinated regulation of C and N metabolisms involving the increase of activities of the three enzymes involved in C metabolism (malate dehydrogenase, isocitrate dehydrogenase, and citrate synthase) as well as the activity of five enzymes involved in N reduction and assimilation (nitrate reductase, nitrite reductase, glutamine synthetase, glutamate synthase, and aspartate aminotransferase) [11,32].

**Figure 5:**
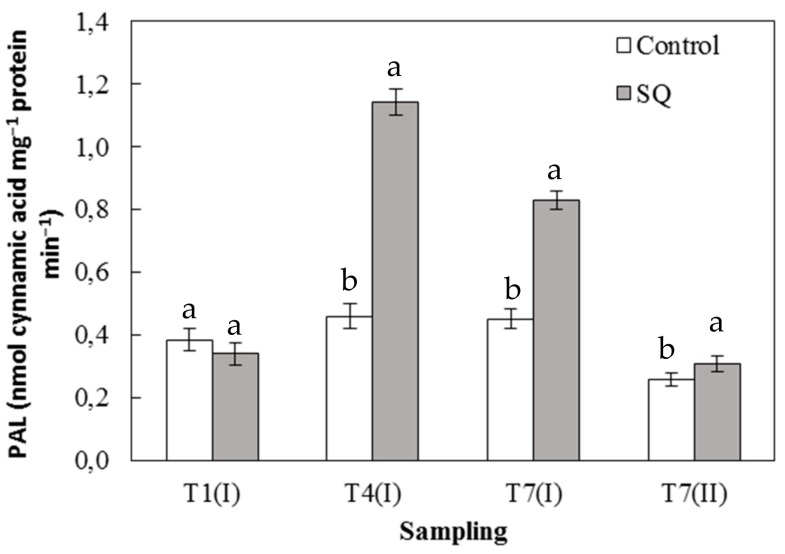
Phenylalanine ammonia lyase (PAL) activity in leaves of lettuce seedlings. Error bars indicate standard deviation. The values are means of data from five replications. Values followed by different letters are significantly different (*p* < 0.05).

Finally, Figure 5 shows the results obtained from the assay of the PAL activity. Similar to the other monitored activities, PAL activity was increased with respect to the control samples, starting from the T4 (I) sampling, in which was recorded the highest increment (around two times) compared to the control (Figure 5). These results show that the treatment with the extract of *S. quadricauda* also determine an induction of secondary metabolism PAL, a key enzyme linking the secondary pathway of phenylpropanoids to the primary metabolism itself. Indeed, it is well known that treatments with algae-based extracts induce the secondary metabolism by activating the biosynthesis pathway of plant defense compounds such as flavonoids and phenylpropanoid [15]. Flavonoids play an important role in plant development and in counteracting the detrimental environmental factors such as high UV light, abiotic and biotic stresses.

These results are very interesting since the treatment, in addition to acting as a biostimulant, by increasing plant growth, inducing protein and chlorophyll accumulation (Table 1, Figure 1 and Figure 2), as well as activating N and C metabolisms (Figure 3 and Figure 4), might also act as a stress modulator, inducing the plant secondary metabolism (Figure 5). All these results, taken together, suggest that algal extracts deeply influence plant physiology by inducing both primary (nitrogen and carbon metabolism) and secondary (PAL) metabolisms. The use of microalgal extract from *S. quadricauda* as a biostimulant in the cultivation of lettuce could replace the use of chemical fertilizers in order to obtain more sustainable cultivation and a reduction of the cost by applying eco-friendly practices.

## 3. Materials and Methods

### 3.1. Microalgae Culture and Extract Preparation

*Scenedesmus quadricauda* (isolated from algal company raceway pond placed in Borculo, Gelderland, Netherland in 2011) was obtained and maintained in the algal collection of Department of Agriculture, Food and Environment (Di3A), University of Catania, Italy. The microalgae was cultivated in a growth chamber using standard BG11 algae culture medium [33] bubbled with air and illuminated by a 3500-lux, average photon flux (PPF) 100 μmol m^−2^ s^−1^ light source (PHILIPS SON-T AGRO 400) with a 12 h photoperiod [34]. The biomass was obtained by centrifugation and the pellet was washed more times with distilled water to reach a conductivity <200 μS cm^−1^ [35].

*S. quadricauda* extract was prepared as described in Barone et al. [19]. In brief, the final microalgal biomass was added to methanol to lyse the cell wall in order to obtain the intracellular contents. After centrifugation and evaporation of the organic solvent, the extract was collected with distilled water to obtain the microalgal extract stock solution.

The characterization of the biomass of S. *quadricauda* and its extract is reported in detail in Barone et al. [19]. In the *S. quadricauda* extract, an increase in alkyl and aromatic carbon, as well as a reduction of N and O alkyl carbon, was observed.

### 3.2. Experimental Conditions 

The experiment was conducted in a transparent container (40 × 20 × 10 cm), filled with an inert substrate such as pumice. The substrate of each container was wetted with 1 L of Hoagland solution: Ca(NO_3_)_2·_4H_2_0, 1180 mg L^−1^; KNO_3_, 505 mg L^−1^; KH_2_PO_4_, 68 mg L^−1^; MgSO_4·_7H_2_0, 493 mg L^−1^; NH_4_NO_3_, 80 mg L^−1^; H_3_BO_3_, 2.86 mg L^−1^; MnCl_2·_4H_2_0, 1.81 mg L^−1^; ZnSO_4·_7H_2_0, 0.22 mg L^−1^; CuSO_4·_5H_2_0, 0.051 mg L^−1^; Na_2_MoO_4_·2H_2_O, 0.12 mg L^−1^; NaFeEDTA, 22.5 mg L^−1^ [36]. Lettuce seedlings (*Lactuca sativa* L.), at four true leaves, were provided by a local nursery in Catania and 10 seedlings were transplanted in each container in a completely random design composed by five replications for treatment, and each replicate was made of 30 seedlings. The seedlings were grown for 6 days in a growth chamber at 25 ± 2 °C, with a 16-h photoperiod and they were irrigated every day with 100 mL distilled water. After this acclimation time (6 days), the first treatment (I) was performed by irrigating the substrate with a solution of Hoagland (500 mL) containing *S. quadricauda* extract to a final concentration of 1 mg Corg L^−1^. This concentration was selected based on previous results obtained on tomatoes [20] and sugar beets [19]. The untreated plants received 500 mL of Hougland solution. The treatment was repeated after 1 week (II). The seedlings were grown for 14 days overall in a growth chamber at 25 ± 2 °C, with a 16-h photoperiod, and they were irrigated every day with 100 mL distilled water.

Four samplings, both in treated (SQ) and untreated plants (control), were performed by randomly picking 5 plants for each replica: T1 (I), after 1 day from the first treatment; T4 (I), after 4 days from the first treatment; T7 (I), after 7 days from the first treatment; T7 (II), after 7 days from the second treatment. Leaf tissues were sampled at each time and immediately frozen with liquid nitrogen and stored at −80 °C until further use.

### 3.3. Physiological Parameters in Lettuce Seedlings

Lettuce seedlings were harvested at each sampling, divided into roots and leaves and separately weighed. Shoot and root lengths were measured with a flexible ruler to the nearest 0.5 mm and the number of leaves for each plant was recorded.

Dry weight was performed for each plant by placing them in a drying oven at 105 °C until constant weight was reached, and allowed to cool for 2 h inside a closed bell jar, then the dry weight of leaves and roots were separately measured.

All measurements were performed on 5 plants for treatment and replicates.

### 3.4. Chlorophyll and Carotenoid Determination

Chlorophylls and carotenoids in lettuce leaves were photometrically determined according to [37,38]. Briefly, plant tissue samples (0.5 g), randomly picked from 5 plants for each replica, were homogenized using 10 mL 80% acetone as the extraction solvent. Samples were centrifuged at 10,000 rpm for 15 min at 4 °C, then an aliquot of supernatant (0.5 mL) was mixed with 4.5 mL of extraction solvent. Chlorophyll and carotenoid contents were measured at three wavelengths, 470, 646.8, and 663.2 nm (Jasco V-530 UV–vis spectrophotometer, Tokyo, Japan), and the relative amount of chlorophyll-a (Ch-a), chlorophyll-b (Ch-b), and carotenoids (C) were calculated as follows and expressed as mg g^−1^ leaf dry weight (DW):

Ch-a = 12.25 A_663.2_ − 279 A_646.8_ (1)

Ch-b = 21.5 A_646.8_ − 5.1 A_663.2_ (2)

C = (1000 A_470_ − 1.82 Ch-a − 85.02 Ch-b)/198 (3)

### 3.5. Protein and Enzyme Extraction from Lettuce Leaves

The extraction of total proteins and enzymes from lettuce leaves was performed according to Lo Piero et al. [39]. Briefly, frozen lettuce leaves were homogenized with an extraction buffer containing 220 mM mannitol, 70 mM sucrose, 1 mM EGTA, 10 mM cysteine, and 5 mM HEPES−KOH pH 7.5 in a 1:1.25 *w*/*v* ratio. The homogenate was filtered and centrifuged at 13,000 rpm for 30 min at 4 °C and the supernatant was recovered and precipitated with solid (NH_4_)_2_SO_4_ at 55% of saturation. The total protein content was determined by the Bradford [40] method, using BSA as a standard curve, and expressed as mg protein g^−1^ DW. Analyses were performed by randomly picking 5 plants for replica.

### 3.6. Enzyme Activities

For each enzymatic activity, an aliquot (1 mL) of the total protein extract, obtained as previously described, was centrifuged at 13,000 rpm for 30 min at 4 °C, the superrnatant was discarded and the pellet was dissolved in the lower volume possible with the appropriate buffer.

Glutamate synthase (GOGAT) activity was performed according to Avila et al. [41]. The assay mixture, in a final volume of 1.1 mL, contained 25 mM Hepes-NaOH (pH 7.5), 2 mM L-glutamine, 1 mM α-ketoglutaric acid, 0.1 mM NADH, 1 mM Na_2_EDTA, and 100 μL of enzyme extract. GOGAT activity was measured spectrophotometrically (Jasco V-530 UV–vis spectrophotometer, Tokyo, Japan) by following NADH oxidation at 340 nm and was expressed as nmol NAD^+^ min^−1^, mg^−1^ protein, using a molar extinction coefficient of 6220 L mol^−1^ cm^−1^.

Glutamine synthetase (GS) was evaluated as a transferase activity, as described in Canovas et al. [42]. The assay mixture was performed in a final volume of 750 μL containing 90 mM imidazole-HCl (pH 7.0), 60 mM hydroxylamine (neutralized), 20 mM KAsO_4_, 3 mM MnCl_2_, 0.4 mM ADP, 120 mM glutamine, and 100 μL of enzyme extract. The enzymatic reaction was conducted for 15 min at 37 °C, then 250 μL of a mixture (1:1:1) of 10% (*w*/*v*) FeCl_3_ ·6H_2_O in 0.2 M HCl, 24% (*w*/*v*) trichloroacetic acid, and 50% (*w*/*v*) HCl were added. The γ-glutamyl hydroxamate produced in the reaction was spectrophotometrically determined at 540 nm and was expressed as μmol γ-glutamyl hydroxamate mg^−1^ protein min^−1^, using a standard curve of γ-glutamyl hydroxamate.

Cytrate synthase (CS) activity was performed as described in Schiavon et al. [11]. The assay mixture, in a final volume of 3 mL, contained 50 μL of 0.17 mM oxalacetic acid, 50 μL of 0.2 mM acetylcoenzyme A (acetyl-CoA), and 100 μL of enzyme extract in 0.1 M Tris-HCl, pH 8.0. The activity was measured spectrophotometrically by monitoring the reduction of acetyl-CoA to CoA at 232 nm, using a molar extinction coefficient of 5400 L mol^−1^ cm^−1^ and was expressed as nmol CoA mg^−1^ protein min^−1^.

Malate dehydrogenase (MDH) activity was performed as described in Schiavon et al. [11]. The assay mixture, in a final volume of 1 mL, was made of 94.6 mM phosphate buffer pH 6.7, 0.2 mM NADH, 0.5 mM oxalacetic acid, 1.67 mM MgCl_2_ and 100 μL of enzyme extract. MDH activity was measured spectrophotometrically by following NADH oxidation at 340 nm and was expressed as nmol NAD^+^ min^−1^, mg^−1^ protein, using a molar extinction coefficient of 6220 L mol^−1^ cm^−1^.

Phenylalanine ammonia lyase (PAL) activity was performed according to Mori et al. [43]. The assay mixture, in a final volume of 1 mL, contained 0.4 mL of 100 mM Tris–HCl buffer (pH 8.8), 0.2 mL of 40 mM phenylalanine, and 200 μL of enzyme extract. The reaction was incubated for 30 min at 37 °C and then stopped with 200 μL of 25% (*v*/*v*) TCA. Samples were centrifuged at 10,000 rpm for 15 min at 4 °C and the absorbance of the supernatant was recorded at 280 nm. PAL activity was expressed as nmol cinnamic acid mg^−1^ protein min^−1^, using a molar extinction coefficient of 16,890 L mol^−1^ cm^−1^.

All enzymatic activities were performed on three replicates for each separate extraction, which was performed on tissues from 5 plants for each treatment and replicates. The protein concentration of each enzyme aliquot was measured by using the Bradford method [40].

### 3.7. Statistical Analysis

Data were analyzed by one-way ANOVA (*p* < 0.05) followed by Tukey’s test for multiple comparison procedures using the statistical software package Statistica v. 13.0 (Dell Inc., Round Rock, TX, USA) to investigate the effect of the treatment on plants. A completely randomized experimental design was adopted, consisting of five plants for each replica.

## 4. Conclusions

This is the first study regarding the effect of *S. quadricauda* extract on the metabolic pathways of lettuce seedlings. Successfully, two weekly treatments positively affected the growth of lettuce seedlings. The morpho-biometric and biochemical parameters of the plants suggest that the *S. quadricauda* extract acts mainly at the leaf level, consequently determining a greater growth at shoot level, mainly due to an increase of dry matter. The positive effect on plant growth is also related to an increase in chlorophyll, carotenoid, and protein contents. From a metabolic point of view, the extract positively affects enzyme activities of GOGAT, CS, and PAL, suggesting that the coordinated mechanism of the regulation of the metabolic pathways of carbon and nitrogen, important for maintaining the balance of the N/C ratio in cells, represents a key point in the mechanism of action of the extract. The extract from *S. quadricauda* rapidly activates the nitrogen and carbon primary metabolisms, producing an increase in plant growth. Furthermore, the treatment positively affects the secondary metabolism by activating the key enzyme of the phenylpropanoid pathway.

Therefore, from the perspective of improved algal biotechnology for a circular economy, the use of a microalgal extract from *S. quadricauda* in the cultivation of lettuce might be an easy and cheaper method to obtain sustainable cultivation of lettuce and a reduction in the cost of chemical fertilizers.

## Figures and Tables

**Table 1:** Morphological traits of lettuce seedlings subjected to *Scenedesmus quadricauda* extract treatment (SQ) at each sampling time (T1 (I), after 1 day from the first treatment; T4 (I), after 4 days from the first treatment; T7 (I), after 7 days from the first treatment; T7 (II), after 7 days from the second treatment). Data are means ± SD. The values are means of data from five plants for each replica. Values in the same column for the same sampling time followed by different letters are significantly different (*p* < 0.05).

Sampling	Treatment	Shoot Height (cm)	Root Length (cm)	Root Weight (g)	Leaves (N°)	Total Plant Weight (g)
T1 (I)	Control	15.00 ± 1.10 a	10.00 ± 1.40 a	1.17 ± 0.21 a	7.00 ± 1.00 a	7.30 ± 0.50 a
	SQ	16.50 ± 0.80 a	11.00 ± 1.00 a	1.86 ± 0.70 a	7.00 ± 1.00 a	8.51 ± 0.52 a
T4 (I)	Control	17.26 ± 0.25 b	9.93 ± 0.40 b	1.59 ± 0.23 a	8.00 ± 1.00 b	7.87 ± 0.60 b
	SQ	19.00 ± 0.20 a	12.17 ± 1.01 a	1.31 ± 0.58 a	10.00 ± 0.00 a	8.75 ± 0.70 a
T7 (I)	Control	18.05 ± 0.78 a	10.00 ± 1.41 b	1.71 ± 0.27 a	11.50 ± 0.71 a	8.47 ± 0.61 b
	SQ	19.00 ± 1.10 a	13.10 ± 1.07 a	1.81 ± 0.30 a	11.50 ± 0.71 a	9.54 ± 0.20 a
T7 (II)	Control	21.10 ± 0.14 a	11.25 ± 1.06 a	1.83 ± 0.16 a	12.50 ± 0.71 a	10.63 ± 0.46 b
	SQ	21.50 ± 1.11 a	10.50 ± 0.71 a	1.82 ± 0.28 a	12.50 ± 0.00 a	12.15 ± 1.00 a

**Table 2:** Chlorophyll and carotenoid contents in leaves of lettuce seedlings subjected to *Scenedesmus quadricauda* extract treatment (SQ) at each sampling time (T1 (I), after 1 day from the first treatment; T4 (I), after 4 days from the first treatment; T7 (I), after 7 days from the first treatment; T7 (II), after 7 days from the second treatment). Ch-a: chlorophylls a; Ch-b: chlorophylls b; C: carotenoids. Data are means ± SD. The values are means of data from five replications. Values in the same column for the same sampling times followed by different letters are significantly different (*p* < 0.05).

Sampling	Treatment	Ch-a (mg g^−1^ DW)	Ch-b (mg g^−1^ DW)	C (mg g^−1^ DW)
T1 (I)	Control	0.323 ± 0.032 b	0.293 ± 0.024 a	0.098 ± 0.014 b
	SQ	0.689 ± 0.020 a	0.295 ± 0.030 a	0.247 ± 0.014 a
T4 (I)	Control	0.405 ± 0.030 b	0.249 ± 0.040 b	0.142 ± 0.018 b
	SQ	0.678 ± 0.030 a	0.388 ± 0.029 a	0.219 ± 0.021 a
T7 (I)	Control	0.546 ± 0.025 b	0.466 ± 0.021 b	0.144 ± 0.020 b
	SQ	0.827 ± 0.042 a	0.583 ± 0.028 a	0.231 ± 0.015 a
T7 (II)	Control	0.463 ± 0.040 b	0.180 ± 0.020 b	0.172 ± 0.018 b
	SQ	0.744 ± 0.025 a	0.286 ± 0.032 a	0.234 ± 0.016 a

## References

[1] Mata T.M., Martins A.A., Caetano N.S. (2010). Microalgae for biodiesel production and other applications: A review. Renew. Sustain. Energy Rev..

[2] Hultberg M., Carlsson A.S., Gustafsson S. (2013). Treatment of drainage solution from hydroponic greenhouse production with microalgae. Bioresour. Technol..

[3] Maurya R., Paliwal C., Chokshi K., Pancha I., Ghosh T., Satpati G.G., Pal R., Ghosh A., Mishra S. (2016). Hydrolysate of lipid extracted microalgal biomass residue: An algal growth promoter and enhancer. Biores. Technol..

[4] Bulgari R., Cocetta G., Trivellini A., Vernieri P., Ferrante A. (2015). Biostimulants and crop responses: A review. Biol. Agric. Hortic..

[5] Parrado J., Bautista J., Romero E.J., García-Martínez A.M., Friaza V., Tejada M. (2008). Production of a carob enzymatic extract: Potential use as a biofertilizer. Biores. Technol..

[6] Ertani A., Pizzeghello D., Baglieri A., Cadili V., Tambone F. (2013). Humic-like substances from agro-industrial residues affect growth and nitrogen assimilation in maize (Zea mays L.) plantlets. J. Geochem. Explor..

[7] Baglieri A., Cadili V., Monterumici C.M., Gennari M., Tabasso S., Montoneri E., Nardi S., Negre M. (2014). Fertilization of bean plants with tomato plants hydrolysates. Effect on biomass production, chlorophyll content and N assimilation. Sci. Hortic..

[8] Alam M.Z., Braun G., Norrie J., Hodges D.M. (2014). Ascophyllum extract application can promote plant growth and root yield in carrot associated with increased root-zone soil microbial activity. Can. J. Plant Sci..

[9] Ertani A., Cavani L., Pizzeghello D., Brandellero E., Altissimo A., Ciavatta C., Nardi S. (2009). Biostimulant activity of two protein hydrolyzates in the growth and nitrogen metabolism of maize seedlings. J. Plant Nutr. Soil Sci..

[10] Sestili F., Rouphael Y., Cardarelli M., Pucci A., Bonini P., Canaguier R., Colla G. (2018). Protein hydrolysate stimulates growth in tomato coupled with N-dependent gene expression involved in N assimilation. Front. Plant Sci..

[11] Schiavon M., Ertani A., Nardi S. (2008). Effects of an alfalfa protein hydrolysate on the gene expression and activity of enzymes of the tricarboxylic acid (TCA) cycle and nitrogen metabolism in Zea mays L.. J. Agric. Food Chem..

[12] Matsumiya Y., Kubo M., El-Shemy H. (2011). Soybean peptide: Novel plant growth promoting peptide from soybean. Soybean and Nutrition.

[13] Colla G., Rouphael Y., Lucini L., Canaguier R., Stefanoni W., Fiorillo A., Cardarelli M. (2016). Protein hydrolysate-based biostimulants: Origin, biological activity and application methods. Acta Hortic..

[14] Rouphael Y., De Micco V., Arena C., Raimondi G., Colla G., De Pascale S. (2017). Effect of Ecklonia maxima seaweed extract on yield, mineral composition, gas exchange and leaf anatomy of zucchini squash grown under saline conditions. J. Appl. Phycol..

[15] Battacharyya D., Babgohari M.Z., Rathor P., Prithiviraj B. (2015). Seaweed extracts as biostimulants in horticulture. Sci. Hortic..

[16] Jannin L., Arkoun M., Etienne P., Laîné P., Goux D., Garnica M., Fuentes M., Francisco S.S., Baigorri R., Cruz F. (2013). Brassica napus growth is promoted by Ascophyllum nodosum (L.) Le Jol. seaweed extract: Microarray analysis and physiological characterization of N, C, and S metabolisms. J. Plant Growth Regul..

[17] Nair P., Kandasamy S., Zhang J., Ji X., Kirby C., Benkel B., Hodges M.D., Critchley A.T., Hiltz D., Prithiviraj B. (2012). Transcriptional and metabolomic analysis of Ascophyllum nodosum mediated freezing tolerance in Arabidopsis thaliana. BMC Genom..

[18] Fan D., Hodges D.M., Critchley A.T., Prithiviraj B. (2013). A commercial extract of brown macroalga (Ascophyllum nodosum) affects yield and the nutritional quality of spinach in vitro. Commun. Soil Sci. Plant Anal..

[19] Barone V., Baglieri A., Stevanato P., Broccanello C., Bertoldo G., Bertaggia M., Cagnin M., Pizzeghello D., Moliterni V.M.C., Mandolino G. (2018). Root morphological and molecular responses induced by microalgae extracts in sugar beet (Beta vulgaris L.). J. Appl. Phycol..

[20] Barone V., Puglisi I., Fragalà F., Stevanato P., Baglieri A. (2019). Effect of living cells of microalgae or their extracts on soil enzyme activities. Arch. Agron. Soil Sci..

[21] Bacellar Mendes L.B., Vermelho A.B. (2013). Allelopathy as a potential strategy to improve microalgae cultivation. Biotechnol. Biofuels.

[22] Barone V., Puglisi I., Fragalà F., Lo Piero A.R., Giuffrida F., Baglieri A. (2019). Novel bioprocess for the cultivation of microalgae in hydroponic growing system of tomato plants. J. Appl. Phycol..

[23] Lucini L., Rouphael Y., Cardarelli M., Canaguier R., Kumar P., Colla G. (2015). The effect of a plant-derived biostimulant on metabolic profiling and crop performance of lettuce grown under saline conditions. Sci. Hortic..

[24] Kopta T., Pavlíková M., Sękara A., Pokluda R., Maršálek B. (2018). Effect of Bacterial-algal Biostimulant on the Yield and Internal Quality of Lettuce (Lactuca sativa L.) Produced for Spring and Summer Crop. Not. Bot. Horti Agrobot..

[25] Spinelli F., Fiori G., Noferini M., Sprocatti M., Costa G. (2010). A novel type of seaweed extract as a natural alter-native to the use of iron chelates in strawberry production. Sci. Hortic..

[26] Taiz L., Zeiger E., Møller I.M., Murphy A. (2014). Plant Physiology and Development.

[27] Yahia E.M., Carrillo-López A., Malda G., Suzán-Azpiri H., Queijeiro Quiroz M. (2018). Photosynthesis. Postharvest Physiology and Biochemistry of Fruits and Vegetables.

[28] Murchie E.H., Pinto M., Horton P. (2009). Agriculture and the new challenges for photosynthesis research. New Phytol..

[29] Lea P.J., Lea P.J., Leegood R.C. (1993). Nitrogen metabolism. Plant Biochemistry and Molecular Biology.

[30] Gupta N., Gupta A.K., Gaur V.S., Kumar A. (2012). Relationship of Nitrogen Use Efficiency with the Activities of Enzymes Involved in Nitrogen Uptake and Assimilation of Finger Millet Genotypes Grown under Different Nitrogen Inputs. Sci. World J..

[31] Alisdair R., Carrari F., Lee J.S. (2004). Respiratory metabolism: Glycolysis, the TCA cycle and mitochondrial elec-tron transport. Curr. Opin. Plant Biol..

[32] Nardi S., Pizzeghello D., Schiavon M., Ertani A. (2016). Plant biostimulants: Physiological responses induced by protein hydrolyzed-based products and humic substances in plant metabolism. Sci. Agric..

[33] Stanier R.Y., Kunisawa R., Mandel M., Cohen-Bazire G. (1971). Purification and properties of unicellular blue-green algae (order Chroococcales). Bacteriol. Rev..

[34] Baglieri A., Sidella S., Barone V., Fragalà F., Silkina A., Nègre M., Gennari M. (2016). Cultivating Chlorella vulgaris and Scenedesmus quadricauda microalgae to degrade inorganic compounds and pesticides in water. Environ. Sci. Pollut. Res..

[35] Puglisi I., Barone V., Sidella S., Coppa M., Broccanello C., Gennari M., Baglieri A. (2018). Biostimulant activity of humic like substances from agro-industrial waste on Chlorella vulgaris and Scenedesmus quadricauda. Eur. J. Phycol..

[36] Armon D.I., Hoagland D.R. (1940). Crop production in artificial culture solutions and in soils with special reference to factors influencing yields and absorption of inorganic nutrients. Soil Sci..

[37] Sumanta N., Haque C.I., Nishika J., Suprakash R. (2014). Spectrophotometric Analysis of Chlorophylls and Carotenoids from Commonly Grown Fern Species by Using Various Extracting Solvents. Res. J. Chem. Sci..

[38] Vanni A., Anfossi L., Cignetti A., Baglieri A., Gennari M. (2006). Degradation of pyrimethanil in soil: Influence of light, oxygen, and microbial activity. J. Environ. Sci. Health B.

[39] Lo Piero A.R., Puglisi I., Petrone G. (2002). Characterization of “Lettucine”, a Serine-like Protease from Lactuca sativa Leaves, as a Novel Enzyme for Milk Clotting. J. Agric. Food Chem..

[40] Bradford M.M. (1976). A rapid and sensitive method for quantitation of microgram quantities of protein utilizing the principle of protein-dye binding. Anal. Biochem..

[41] Avila C., Rotella J.R., Canovas F.M., De Castro I.N., Valpuesta V. (1987). Different characteristics of the two glutamate synthetases in green leaves of Lycopersicon esculentum. Plant Physiol..

[42] Canovas F.M., Canton F.R., Gallardo F., Garcia-Gutierrez A., de Vincente A. (1991). Accumulation of glutamine synthetase during early development of maritime pine (Pinus pinaster) seedlings. Planta.

[43] Mori T., Sakurai M., Sakuta M. (2001). Effects of conditioned medium on activities of PAL, CHS, DAHP synthase (DS-Co and DS-Mn) and anthocyanin production in suspension cultures of Fragaria ananassa. Plant Sci..

